# Letter to Dobesh et al. on lower mortality with andexanet alfa in factor Xa inhibitor–related major bleeding

**DOI:** 10.1016/j.rpth.2024.102428

**Published:** 2024-04-27

**Authors:** Stefani Lucarelli, Katrina Derry, Steven Atallah, Craig Stevens

**Affiliations:** 1 Department of Pharmacy, UC San Diego Health, San Diego, California, USA; 2 UC Health, University of California Office of the President, Oakland, California, USA; 3 Department of Pharmacy, UC Irvine Health, Orange, California, USA

To the Editor,

The recent publication in Research and Practice in Thrombosis and Haemostasis by Dobesh et al. [1] reported that treatment with andexanet for factor (F)Xa inhibitor–associated bleeding resulted in 50% lower odds of in-hospital mortality compared with 4-factor prothrombin complex concentrate (4F-PCC). We aim to raise methodological concerns and highlight key misconceptions of this retrospective cohort study that are inconsistent with other literature.

The controversy of agent selection when treating FXa-associated bleeding is well known. Andexanet Alfa for Acute Major Bleeding Associated with Factor Xa Inhibitors studied andexanet use in FXa-associated major bleeds and excluded the sickest patients: those with expected survival of <1 month, intracranial hemorrhage with Glasgow Coma Scale (GCS) score of <7, and hematoma volume of >60 mL. The investigators’ study population is similar to Andexanet Alfa for Acute Major Bleeding Associated with Factor Xa Inhibitors in which they explore major, but not emergent, life-threatening bleeding as door-to-needle time averaged approximately 8 hours. Mechanistically, 4F-PCC provides exogenous coagulation factors, whereas andexanet temporarily and reversibly inhibits FXa inhibitors. In patients with larger bleeds or higher expected overall mortality, 4F-PCC may be deemed more appropriate due to its mechanism. Thus, clinicians may allocate—or channel—patients with poor predicted outcomes to the 4F-PCC group.

The investigators did not provide a statistical comparison of baseline patient characteristics, several of which may suggest that the 4F-PCC arm was more critically ill at baseline. As noted in Table 4 by Dobesh et al. [1], a higher percentage of 4F-PCC patients had altered mental status (37.1% vs 33.2%), multicompartment intracranial hemorrhages (9.1% vs 6.2%), trauma-induced bleeds (55.3% vs 49.1%), GCS score of ≤8 (35.2% vs 30.8%), and larger hematoma volumes (median [IQR], 22.5 [10.0-38.8] vs 14.5 [5.8-30.0]). Data on GCS and hematoma volume were underreported, both of which are known predictors of mortality in intracranial hemorrhage [2]. Severe GCS (vs mild) was a significant predictor of mortality regardless of treatment (odds ratio, 48.04; 95% CI, 22.91-115.11; *P* < .01). This absolute difference of 4.4% may in part explain the absolute difference in mortality of 4.6%.

Patients who received both andexanet and 4F-PCC were excluded. Based on the mechanisms of bleeding and treatment options, a provider may be compelled to reach for 4F-PCC in a decompensating patient who received andexanet, thereby excluding them from the trial. The inverse (ie, patients receiving 4F-PCC who decompensate and receive andexanet) may be uncommon, causing poor 4F-PCC patient outcomes to remain included. Patients who received 4F-PCC and required a second 4F-PCC dose remained included and represented 6.2% (*n* = 135) of that treatment arm.

Differences in baseline characteristics and methodological concerns may have resulted in higher mortality rates among the 4F-PCC cohort. The unpublished results of the highly anticipated prospective randomized ANEXA-I trial, which compares andexanet with standard of care (in which 86.9% of patients received 4F-PCC), showed no difference in mortality and numerically higher mortality in the andexanet arm (27.8% vs 25.5%) [3]. The relative reduction in mortality seen by Dobesh et al. [1] must be juxtaposed with the relative differences in baseline characteristics that may explain the higher mortality risk in the 4F-PCC arm. Due to potential channeling bias, a possibly impactful exclusion criterion, and failure to replicate these results in prospective trials, we hesitate to use these data to influence prescribing practices in our patients.
